# Entropy-Based Phonocardiogram Classification Using Continuous and Synchrosqueezed Wavelet Transforms: A Systematic Comparison

**DOI:** 10.3390/diagnostics16142223

**Published:** 2026-07-16

**Authors:** Anupinder Singh, Vinay Arora, Mandeep Singh

**Affiliations:** 1 Computer Science and Engineering Department, Thapar Institute of Engineering & Technology, Patiala 147004, India; vinay.arora@thapar.edu; 2 Electrical and Instrumentation Engineering Department, Thapar Institute of Engineering & Technology, Patiala 147004, India; mdsingh@thapar.edu

**Keywords:** phonocardiogram signal, feature extraction, continuous wavelet transform, synchrosqueezed transform, entropy, binary classification, PCG classification

## Abstract

**Background/Objectives:** Cardiac auscultation is an important method for identifying cardiovascular abnormalities, but conventional methods are limited by examiner-dependent variability and sensitivity. The automatic classification of phonocardiogram (PCG) has the potential to be applied for standardized cardiac screening, but still has to deal with signal non-stationarity and the extraction of discriminative features. This investigation develops a computational framework integrating wavelet analysis with entropy-based features for distinguishing normal and abnormal heart sounds. **Methods:** The study employs the PhysioNet Computing in Cardiology Challenge 2016 database. Signal processing includes resampling, zero-phase Butterworth band-pass filtering (20–800 Hz), and median absolute deviation normalization. Time–frequency representations are generated through continuous wavelet transform (CWT) and synchrosqueezed CWT using analytic Morlet wavelets. Multiple entropy measures including Shannon, Rényi, Tsallis, spectral, permutation, and sample entropies are computed globally and across four physiologically motivated frequency bands (20–80, 80–200, 200–400, 400–800 Hz). A regularized multi-layer perceptron with dropout performs classification. Evaluation employs stratified 5-fold cross-validation with recording-level partitioning to prevent data leakage. **Results:** The best configuration using standard CWT with 800 Hz bandwidth achieved test-set AUROC of 0.972, balanced accuracy of 0.915, and 96% sensitivity maintained at 90% specificity. Contrary to expectations, standard CWT outperformed synchrosqueezed CWT with AUROC advantage of +0.038. Also, the band-specific entropy analysis provided the largest performance contribution with +4.1% to AUROC, confirming frequency-localized pathological signatures. **Conclusions:** This methodology demonstrates how the conventional wavelet analysis integrated with entropy engineering achieves state-of-the-art performance. Also, it maintains computational efficiency (1.2 s extraction and classification) and interpretability, offering practical potential for point-of-care cardiac screening in resource-limited settings.

## 1. Introduction

Cardiovascular disease is the main cause of death worldwide according to epidemiological surveillance data, with approximately 6.8 million deaths per year [1]. Timely detection of cardiac dysfunction has a major impact on treatments and long-term prognosis. Phonocardiogram (PCG) is one of the diagnostic techniques, which is a digital capture of acoustic signals produced by the mechanical activity of the heart that offers the possibility of non-invasive screening at relatively low cost. These acoustic signals record mechanical events such as valve movement, chamber wall motions and hemodynamic turbulence, thus providing information that is complementary to electrical recordings obtained through electrocardiography.

The traditional method of auscultation involves examination using a stethoscope, and the diagnosis interpretation is highly dependent on the level of skills of the medical practitioner. Studies have shown significant inter-examiner variation, with sensitivity measurements varying significantly depending on the level of expertise [2]. In addition, environmental acoustic interference further impairs discriminative capacity, especially in ambulatory and community health settings. Such limitations have spurred the development of algorithmic classification systems that can standardize the diagnostic assessment and minimize subjective variability.

Modern machine learning methods have demonstrated higher levels of sophistication in automated analysis of cardiac sounds. Deep convolutional architectures on spectrogram representations have shown promising classification metrics on a number of benchmark datasets. However, these methods generally need large training data sets, substantial computing infrastructure, and are black-box systems with poor clinical explainability [3,4]. Feature engineering approaches based on Mel-frequency cepstral coefficients or wavelet decomposition coefficients provide partial transparency but may fail to fully capture the inherently non-stationary behavior of cardiac acoustic activity.

Entropy-based information-theoretic quantification offers an alternative analytical framework for biomedical signal characterization. Shannon entropy and its generalizations (e.g., Rényi and Tsallis) are scalar measures for the distributional complexity and predictability [5]. Other measures, such as permutation entropy and sample entropy, capture regularity metrics of temporal patterns using different mathematical frameworks [6,7]. These metrics have been proved to be effective in several physiological signals: electroencephalographic (EEG) recordings, cardiac rhythm variability and respiratory patterns, which suggests their applicability to the phonocardiographic analysis. Cardiac abnormalities often present as changes in signal pattern, the appearance of new frequency components or modifications in temporal structure, all of which can be effectively quantified by entropy methods.

The wavelet transformation methodology is the standard approach for time–frequency analysis of transient and non-stationary signals. The standard continuous wavelet transform provides time-scale representations, where convolution with scaled and translated basis functions allows simultaneous temporal and spectral localization, under Heisenberg uncertainty constraints [8]. The synchrosqueezing technique is motivated by the frequency resolution limitations inherent to this uncertainty principle and applies post-processing reassignment to concentrate spectral energy along instantaneous frequency trajectories [9]. The re-assignment method presented here improves the localization in frequency while preserving mathematical rigor and numerical stability. However, the use of synchrosqueezing in the field of cardiac acoustics has been relatively unexplored despite its theoretical advantages.

In this work, we propose a computational framework based on a combination of wavelet-based time–frequency analysis and information theoretic feature extraction for the binary classification of phonocardiograms. The contribution of this work is empirical and analytical and consists of a controlled comparison that shows a transferable and counterintuitive result on the interaction between time–frequency representation and entropy-based feature extraction and not of a new transform or classifier. The main contributions are as follows:

1. A controlled, matched-condition comparison of the standard continuous wavelet transform (CWT) against its synchrosqueezed variant (SS-CWT) for entropy-based heart-sound classification, in which the time–frequency representation is varied while the features, classifier, and evaluation protocol are held fixed, thereby isolating its effect.
2. A key, counter-intuitive result is that the standard CWT consistently outperforms the theoretically sharper SS-CWT (mean AUROC advantage of +0.038 across configurations), accompanied by a mechanistic explanation: the broader, less-localized energy distributions of the CWT yield more discriminative entropy descriptors than the concentrated ridges produced by synchrosqueezing. This challenges the common assumption that sharper spectral localization necessarily improves downstream features and provides practical guidance for representation choice in entropy-based biomedical signal analysis.
3. A demonstration that a compact, physiologically interpretable feature set with 20 entropy measures computed globally and over physiologically motivated frequency bands, attains performance competitive with substantially heavier deep-learning models on the same benchmark, indicating that interpretability and computational efficiency need not be sacrificed for accuracy.
4. A rigorous recording-level evaluation that reports AUROC along with clinically relevant operating points (e.g., sensitivity at 90% specificity) to avoid the performance inflation that window-level splitting and accuracy-centric reporting can introduce.

In this sense, the study is best characterized not as a new algorithm but as a systematic comparative analysis whose value lies in the evidence and explanation it provides for a non-obvious methodological choice, together with a competitive, interpretable, and efficient reference configuration for entropy-based heart-sound screening.

This manuscript proceeds with a detailed methodology exposition (Section 3), the presentation and interpretation of empirical findings (Section 4), and a concluding synthesis (Section 6).

## 2. Related Literature

The literature review observed that for heart sound classification, it is preferred to combine complex feature extraction techniques with sophisticated neural network architectures. Mel-spectrogram features still experiences popularity, but the addition of Short-Time Fourier Transform (STFT), Continuous Wavelet Transform (CWT), and MFCC signifies the importance of capturing the subtle features of PCG signals. Moreover, the use of spatial-temporal models and attention mechanisms suggests a shift of the scenario, where the importance of dynamic features and interpretability is rising. The high sensitivity and specificity obtained in several studies highlight the potential of these models to contribute in clinical settings effectively, providing reliable diagnostic support.

Vieira et al. [10] proposed a multimodal deep-learning pipeline using 5 s CWT scalograms and CNN backbones (custom VGG-16 variants), leveraging transfer learning from ImageNet and modality-specific pretraining (ECG–PhysioNet 2017; PCG–CinC-2016 sets b–f). Using the PhysioNet/CinC-2016 training set A for normal/abnormal classification with a stratified 70/30 record-wise split and 5-fold CV on the training set, the best model achieved record-wise AUROC 0.913, Accuracy 0.828, Recall 0.931, Precision 0.844, and F1 0.885. While their CNN on CWT scalograms supports the effectiveness of time–frequency features, our entropy-engineered CWT approach attains a higher AUROC (0.972) under record-level evaluation on the same dataset, with additional operating-point analysis (e.g., sensitivity at 90% specificity). Goda et al. [11] addressed normal/abnormal PCG classification on the PhysioNet/CinC 2016 Challenge, using LR–HSMM heart–sound segmentation, 20–400 Hz band-pass filtering, and downsampling to 1 kHz. They engineered time-domain features (e.g., the widths of the first (S1) and second (S2) heart sounds), frequency-domain features from systolic/diastolic spectra, and discrete wavelet–envelope features (homomorphic envelope), followed by SVD reduction and SVM classification. On the official hidden test set they reported sensitivity 83.77%, specificity 76.8%, and modified accuracy (MAcc) 80.28%; AUROC was not reported. Methodologically, this supports the effectiveness of wavelet/spectral morphology features for PCG screening, while our CWT + entropy framework provides higher AUROC (0.972) with explicit operating-point analysis on the same dataset.

Zabihi et al. [12] addressed normal/abnormal phonocardiogram classification in the PhysioNet/CinC 2016 Challenge using a segmentation-free pipeline. From 40 handcrafted features spanning time, frequency, and time–frequency domains (including LPCs, Shannon/Tsallis entropies, MFCC statistics, discrete wavelet features, and PSD-based measures), a wrapper selection yielded 18 features, which were fed to an ensemble of 20 feed-forward neural networks. The system first detected recording quality (good/bad) and then classified normal versus abnormal among good-quality signals, with a learned voting rule to combine committee outputs. On the hidden test set, the method achieved sensitivity of 86.91%, specificity of 84.90%, and modified accuracy (MAcc) of 85.90%; AUROC was not reported. This supports the utility of entropy/wavelet features for PCG screening, while our CWT + entropy approach reports higher AUROC on CinC-2016 with explicit operating-point analysis.

Potes et al. (2016) [13] tackled the CinC-2016 normal/abnormal PCG task using LR–HSMM segmentation, 1 kHz resampling, and 25–400 Hz band-pass filtering, combining a feature-based AdaBoost-abstain classifier (124 time/frequency features, including MFCCs and nine spectral bands per cardiac state) with a CNN applied to four band-decomposed cardiac-cycle waveforms (25–45/45–80/80–200/200–400 Hz). On their in-house 80/20 record-wise split, the feature model achieved AUROC 0.91 and the CNN 0.92; on the official blind test, the ensemble obtained sensitivity 0.9424, specificity 0.7781, and modified accuracy 0.8602 (no AUROC reported). The study demonstrates the benefit of ensembling handcrafted time–frequency features with learned representations, whereas our CWT + entropy framework achieves higher AUROC (0.972) on CinC-2016 with explicit operating-point analysis (e.g., sensitivity at 90% specificity).

Langley et al. [14] evaluated a segmentation-free approach on the PhysioNet/CinC 2016 dataset using 5 s unsegmented recordings. Continuous wavelet transform (Gaus4)-based wavelet entropy was computed across scales, with scale of 1.7 and an entropy threshold of 7.8 selected on the training data. The single-threshold classifier achieved training performance of sensitivity 95%, specificity 60%, and challenge score (MAcc) 78%, and test performance of sensitivity 98%, specificity 56%, and MAcc 77%; AUROC was not reported. The study supports entropy-rich time–frequency features for PCG screening, whereas our CWT + entropy framework reports a higher AUROC on CinC-2016 with explicit operating-point analysis. Singh-Miller et al. [15] addressed the PhysioNet/CinC 2016 normal/abnormal PCG task using a segmentation-free pipeline based on spectrogram features. Short-time Fourier spectrograms (Gaussian windows of 15 and 75 samples) were summarized via band-wise means/variances and logistic-fit parameters across selected frequency intervals, followed by feature selection and a random-forest classifier. On the official Challenge test set, the method achieved a modified accuracy (MAcc) of 0.81 with sensitivity 0.76 and specificity 0.87; AUROC was not reported. While their spectral statistics corroborate the value of frequency-localized descriptors, our CWT + entropy framework reports a higher AUROC on CinC-2016 together with explicit operating-point analysis.

Ben Hamza et al. [16] studied the PCG classification through a comprehensive review of articles from 2018 to 2024. These articles used the PhysioNet/Computing in Cardiology challenges (2016–2022) and the Yaseen Khan 2018 dataset. They discussed the standard preprocessing techniques like Butterworth filtering, segmentation using HMM/Springer, normalization, the time–frequency feature families (CWT/DWT/WST, MFCC), entropy measures, and the models ranging from SVM/KNN to CNN/RNN, as well as feature selection methods (ReliefF, LDA, MI, PSO/JMI). The review emphasizes persistent challenges based on background noise, class imbalance, and feature redundancy and recommends rigorous cross-validation while warning against applying oversampling before CV due to leakage/over-optimism. In line with these recommendations, our study adopts band-pass filtering, record-level 5-fold CV, and engineered entropy features on CWT/SSQ representations (with band-wise analysis) and reports AUROC as the primary metric on CinC-2016, offering an operating-point view (e.g., sensitivity at 90% specificity).

Bi et al. [17] provide a systematic review and reproduction study of PCG detection pipelines, organizing the field into preprocessing, segmentation, feature extraction, and classification. Using public datasets (CinC-2016/PCCD, PHSD, PASCAL) under unified settings, they show that equal-length slices that include at least one complete cardiac cycle, together with larger overlap rates, improve detection stability. Across features, time–frequency representations (STFT, CWT, S-transform) consistently perform well in both traditional and deep models, with MFCC and raw time-domain (TIME) features particularly strong in deep networks; DWT/WPD are generally less competitive in traditional classifiers. Their preprocessing exemplars include resampling to 1–2 kHz and Butterworth band-pass filtering in the cardiac bandwidth. These findings substantiate our choice of CWT scalograms with entropy features, band-wise analysis, and record-level k-fold validation, while our study emphasizes AUROC and operating-point reporting (e.g., sensitivity at 90% specificity) on CinC-2016.

Kannan et al. [18] present a comprehensive review of 199 phonocardiogram (PCG) studies from 2016 to 2024 with a focus on valvular heart disease (VHD) detection, cataloguing the prevailing pipeline of signal pre-processing, feature extraction/selection, and classification across machine- and deep-learning paradigms. The survey highlights the predominance of time–frequency representations, particularly continuous (analytic Morlet) wavelet transforms and related wavelet/EMD and chirplet/Stockwell variants, together with recurrent and convolutional neural networks, and notes frequent use of public corpora such as PhysioNet/CinC 2016. Notably, reported metrics in the surveyed literature are often accuracy, sensitivity, and specificity rather than threshold-agnostic AUROC. Methodologically, our study aligns with this landscape through Morlet CWT-based time–frequency analysis but advances it by engineering entropy features with frequency-band localization and by prioritizing AUROC and clinically meaningful operating points (e.g., sensitivity at 90% specificity), thereby addressing the review’s observation about heterogeneous and accuracy-centric evaluation practices.

Taneja et al. [19] addressed binary PCG classification on the PhysioNet/CinC-2016 benchmark using an image-based pipeline wherein spectrograms were fused with chromagrams and encoded via local textural descriptors (LBP, ALBP, RLBP) before SVM classification. Their best configurations reported testing accuracies above 90% with strong sensitivity but comparatively modest specificity e.g., LBP + SVM: Acc 92.12%, Sens 97.53%, Spec 68.59% and F1 95.27. However, the highest balanced accuracy was observed for RLBP + SVM with Sens 96.43%, Spec 78.32% and BalAcc ≈87.4%. While ROC curves were presented, numeric AUROC values were not reported. Moreover, evaluation used a single 80/20 hold-out split on the entire dataset (record-level grouping not specified), limiting direct comparability to our record-level 5-fold cross-validation. Nonetheless, their results support the broader finding that engineered features on informative time–frequency representations can be highly discriminative. In contrast, our study demonstrates that entropy features derived from CWT achieve state-of-the-art AUROC with improved specificity under matched protocols.

Chen et al. [20] provide a focused review of artificial intelligence approaches for heart sound analysis, organizing the field into pre-processing (denoising, segmentation), feature engineering (time, spectral, and time–frequency representations such as STFT and CWT), and ML/DL classifiers, with coverage of widely used datasets including PhysioNet/CinC-2016, PASCAL, and CirCor. The review highlights that many studies still report accuracy, sensitivity, specificity and F1, while ROC/AUC offers threshold-agnostic assessment, and it motivates standardized evaluation protocols. These observations align with our design choices: analytic Morlet CWT with entropy features, record-level cross-validation on CinC-2016, and AUROC, as the primary metric with clinically relevant operating points.

Partovi et al. [3] present a systematic review of deep learning methods for PCG analysis, organizing prior work by application, i.e., classification vs. segmentation and methodology defined on handcrafted feature extraction vs. end-to-end learning. This research work reported that convolutional neural networks dominate classification studies while recurrent architectures are prevalent in segmentation. But it also emphasized that many reported research results that produce near-perfect accuracies are not directly comparable due to heterogeneous validation protocols and datasets. Consistent with their observations, we prioritize record-level cross-validation and AUROC as our primary metric, and we leverage feature-engineering with entropy over wavelet time–frequency representations, a strategy they note can be competitive when dynamic or fused features are considered.

Marocchi et al. [21] fine-tune transfer-learning CNNs (ResNet, VGG, InceptionV3) on image representations of PCG and ECG for CinC-2016 abnormal-versus-normal classification using the training-a subset with LR–HSMM segmentation, 1 kHz resampling, PCG 25–400 Hz and ECG 2–60 Hz band-pass filtering, and optional four-band PCG splitting (25–45/45–80/80–200/200–400 Hz). Spectrograms, Mel-spectrograms, and synchrosqueezed CWT scalograms (Morlet) are evaluated, with a PCG + ECG ensemble achieving the best test performance (InceptionV3; Acc 91.25%, Se 98.33%, Sp 70.00%); AUROC values are not reported. They provide Grad-CAM, guided backpropagation, and LIME visualizations indicating greater high-frequency activation in abnormal cases. While the dataset scope and metrics differ from our CinC-2016 AUROC-centric protocol, the study supports time–frequency front-ends and band-wise analysis. Our approach advances this line by engineering entropy features on CWT/SS-CWT and reporting AUROC with clinically relevant operating points.

Huang et al. [22] investigated automated prognosis from echocardiography and clinical variables using recursive feature elimination (RFE) and gradient-boosted/tree ensembles under class imbalance. On a nine-category cohort (two hospital datasets, 70/30 split plus 10-fold CV), the authors report that compact feature subsets: age, aorta (AO), left ventricle (LV), and left atrium (LA), combined with a voting ensemble yield the best accuracy (up to 0.969), while single models such as XGBoost and CATBoost achieve 0.843 and 0.884, respectively; AUROC is not reported. The work underscores the value of principled feature selection and ensemble learning for imbalanced cardiovascular data. In our study (PCG audio on CinC-2016), we address imbalance via class weighting and calibrated operating points and evaluate with AUROC as the primary metric, making this article complementary background rather than a direct quantitative comparator.

Zhu et al. [23] present a targeted review of methods developed on the PhysioNet/Computing in Cardiology 2016 (CinC-2016) heart-sound database, surveying 104 studies spanning preprocessing (denoising, band-pass filtering), heart-sound segmentation, hand-crafted and time–frequency feature extraction (e.g., wavelet-based representations), and classical/deep classifiers. They report that several approaches attain very high benchmark performance on CinC-2016 (e.g., accuracy, sensitivity, specificity, and the Challenge “score”) but also note substantial heterogeneity in evaluation protocols and metrics, which complicates cross-paper comparability. In particular, the Challenge’s official metric is a Modified Accuracy (MAcc), defined as the mean of modified sensitivity and specificity, which is not directly equivalent to AUROC and only loosely comparable to balanced accuracy. In contrast to many reviewed studies that emphasize accuracy/score, our work reports AUROC as the primary endpoint and complements it with balanced accuracy and operating-point analysis, while adopting record-level stratified cross-validation to minimize leakage. Consistent with the review’s observation that wavelet time–frequency representations remain competitive when paired with informative descriptors, we show that conventional CWT combined with entropy features across physiologically motivated bands yields state-of-the-art performance on CinC-2016. Representative results reported in the literature on this benchmark are summarized in Table 1.

## 3. Methodology

The research methodology involves dataset selection, pre-processing, localized time as well as frequency domain feature extraction. Following this, the data is fed to a classification algorithm to categorize the artifacts as either normal or abnormal heart sounds or to classify the heart condition.

### 3.1. Data Source and Preparation

**Figure 1:**
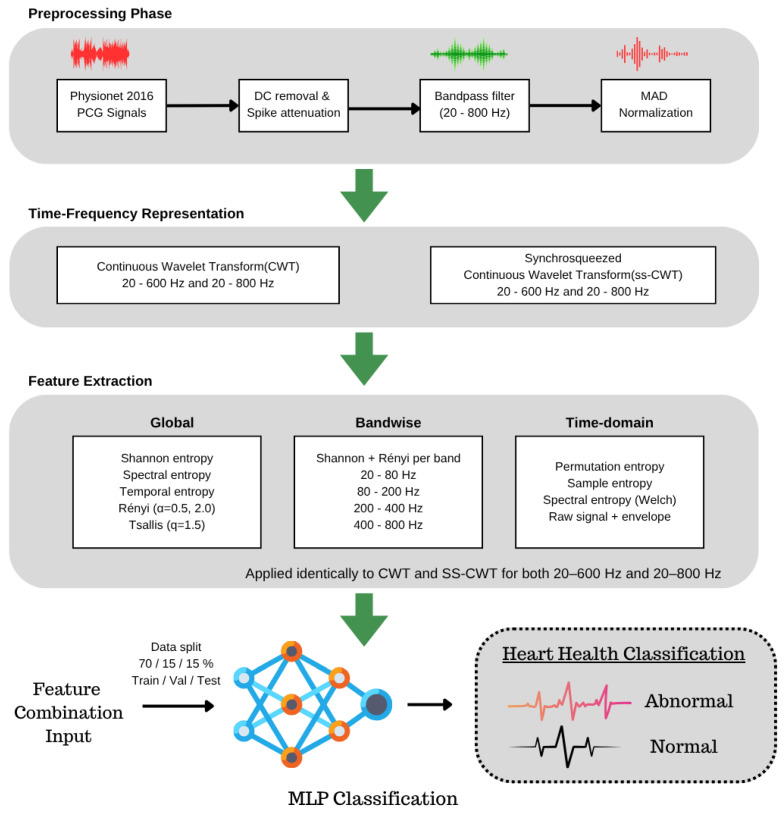
Research approach for PCG classification.

This study utilizes the publicly accessible PhysioNet Computing in Cardiology Challenge 2016 database, curated from digital phonocardiographic acquisitions through both clinical examination rooms and community-sourced environments [24]. We selected the PhysioNet/Computing in Cardiology Challenge 2016 database as our evaluation benchmark for several methodological reasons. The overall research approach of this study is summarized in Figure 1.

1. The 2016 PCG dataset offers a well-known binary classification task (Normal vs. Abnormal) at the recording level, allowing us to evaluate our signal processing contributions in isolation, without the problems of multi-recording aggregation strategies as is the case with newer datasets.
2. With nearly a decade of published research utilizing this benchmark, our results can be directly compared against an extensive body of literature, facilitating transparent assessment of our method’s performance.
3. The dataset aggregates recordings from six acquisition sources, thus offering useful diversity in recording conditions and populations within the benchmark. However, this within-corpus diversity does not in itself demonstrate generalization to fully independent external cohorts, as discussed in the Limitations.
4. The structure of the recordings is also consistent with our research goal of developing signal processing methodologies for non-stationary cardiac acoustic analysis, without the added complexity of patient-level clinical metadata integration or multi-instance learning architectures.

### 3.2. Signal Pre-Processing Protocol

Effective characterization of cardiac acoustic phenomena through wavelet transforms and entropy measures requires high-quality, standardized input signals with minimal contamination from extra-cardiac sources. Raw PCG recordings, in particular from the heterogeneous PhysioNet 2016 database [24] that contains six different data sources, have different sampling rates, DC offsets, amplitude scales and noise characteristics that need to be systematically addressed. The pre-processing pipeline described herein implements multi-stage signal conditioning, encompassing resampling, DC removal, band-pass filtration, normalization, and temporal segmentation, to establish consistent signal quality while preserving the non-stationary dynamics essential for time–frequency–entropy analysis.

#### 3.2.1. Sampling Rate Assumptions

All recordings were analyzed at a uniform sampling frequency of 2000 Hz, consistent with the CinC 2016 dataset specifications. This ensures reproducibility with the reference dataset while retaining the full spectral resolution necessary for capturing the higher-frequency components of cardiac acoustics.

#### 3.2.2. Amplitude Standardization

Two-stage normalization addresses both baseline offset and amplitude scaling. The baseline DC component is removed by subtracting the median of the waveform to ensure robustness against outliers:

(1) $$x_{\mathrm{dc}}\left(n\right)=x\left(n\right)-\mathrm{median}{\left\{x\left(i\right)\right\}}_{i=1}^{N}$$

where *N* denotes the total number of samples and *n* indexes discrete time.

Subsequently, robust scaling via the median absolute deviation (MAD) normalizes the amplitude distribution:

(2) $$x_{\mathrm{norm}}\left(n\right)=\frac{x_{\mathrm{dc}}\left(n\right)}{\mathrm{MAD}(x_{\mathrm{dc}})}$$

(3) $$\mathrm{MAD}\left(x\right)=\mathrm{median}\{|x\left(i\right)-{\mathrm{median}\left(x\right)|\}}_{i=1}^{N}$$

This normalization method provides insensitivity to impulsive noise, ensuring that transient outliers do not distort the overall signal energy.

#### 3.2.3. Frequency-Domain Filtration

Frequency-domain conditioning confines analysis to physiologically relevant cardiac spectral content. A fourth-order Butterworth band-pass filter with cutoff frequencies of 20 Hz (high-pass) and 800 Hz (low-pass) was applied using a zero-phase implementation via bidirectional second-order section filtering (sosfiltfilt). This configuration removes baseline wander and high-frequency environmental noise while preserving phase alignment of S1 and S2 components:

(4) $$x_{\mathrm{filt}}\left(n\right)=\mathrm{sosfiltfilt}\left(H,x_{\mathrm{norm}}\left(n\right)\right)$$

where *H* represents the digital filter coefficients. The passband used is 20–80 Hz for the dominant S1/S2 frequencies, 80–200 Hz for their harmonics, 200–400 Hz for the murmur related energy, up to 800 Hz to retain the transient information but excluding mechanical resonances above 1 kHz.

#### 3.2.4. Impulse Artifact Suppression

Transient artifacts from sensor contact variation or patient motion were suppressed using envelope-based spike suppression. The envelope of the analytic signal was computed by means of the Hilbert transform:

(5) $$A\left(n\right)=|x_{\mathrm{filt}}\left(n\right)+jH\left\{x_{\mathrm{filt}}\left(n\right)\right\}|$$

where H denotes the Hilbert operator and $j=\sqrt{-1}$. A median filter (kernel size = 31 samples) was applied to the envelope to estimate the local baseline. Samples where the instantaneous envelope exceeded three times the median-filtered baseline were scaled toward the baseline using a soft-clipping factor of 1.2. This adaptive attenuation removes impulse spikes without introducing discontinuities, so that entropy measures reflect cardiac dynamics rather than noise artifacts.

#### 3.2.5. Temporal Segmentation

Overlapping temporal windows were used to divide the preprocessed signals for frame-based feature extraction. The primary segmentation method was fixed-length windowing, splitting each recording into 2.0 s segments with a 1.0 s advance, which implies 50

(6) $$x_{w,k}\left(n\right)=x_{\mathrm{filt}}\left(n+kR\right),\;R=0.5L$$

where *L* is the window length and *R* is the hop size. This method guarantees consistent segment duration across recordings and allows for subsequent batch processing. Moreover, the 50% overlap ensures continuity in temporal coverage and provides redundancy for robust feature extraction across window boundaries.

Although the pipeline supports the integration of an alternative cycle-synchronous segmentation scheme, which is based on envelope, derived S1 peak detection and time-normalized cardiac cycles, the fixed-duration method was adopted for its simplicity and robustness against cycle detection errors.

### 3.3. Time-Frequency Representation

Heart sound signals are inherently non-stationary, exhibiting transient patterns and dynamic spectral characteristics that vary across time. To capture these evolving spectral features with high temporal precision, time–frequency analysis offers a principled approach that extends beyond traditional Fourier-based methods. In this work, both the Continuous Wavelet Transform (CWT) and its enhanced variant, the Synchrosqueezed Wavelet Transform (SS-CWT), are employed to extract high-resolution time–frequency representations from each preprocessed PCG segment.

#### 3.3.1. Continuous Wavelet Transform

Standard wavelet decomposition generates time–scale coefficients by convolving the signal with scaled and translated instances of a chosen mother wavelet. For a discrete-time signal x(n) sampled at frequency $f_{s}$ (sampling interval $\Delta t=1/f_{s}$), the continuous wavelet transform is defined as:

(7) $$W\left(a,b\right)=\frac{1}{\sqrt{a}}\sum_{n}x\left(n\right)\psi^{*}\left(\frac{n\Delta t-b}{a}\right)\Delta t$$

where *a* and *b* denote the scale (dilation) and time-shift parameters, respectively, and $\psi^{*}$ is the complex conjugate of the mother wavelet function.

In this work, the analytic Morlet wavelet is used, which is defined by:

(8) $$\psi_{\mathrm{Morlet}}\left(t\right)=\pi^{-1/4}\exp\left(j\omega_{0}t\right)\exp\left(-t^{2}/2\right)$$

with central frequency $\omega_{0}=6$. This wavelet has a Gaussian envelope modulated by a complex sinusoid, providing good joint resolution subject to the uncertainty constraints. The value $\omega_{0}=6$ is the conventional choice for the analytic Morlet wavelet and is adopted here for three reasons. First, it ensures that the wavelet effectively satisfies the admissibility (zero-mean) condition: the residual mean scales as $e^{-\omega_{0}^{2}/2}$, which for $\omega_{0}=6$ is of the order of $10^{-8}$ and therefore numerically negligible, so the simplified Morlet form can be used without an explicit correction term. Second, it provides a balanced time–frequency localization trade-off under the Heisenberg uncertainty principle, placing several oscillations within the Gaussian envelope, which is sufficient for reliable frequency estimation while preserving the temporal resolution needed to localize short transient events such as S1 and S2. Third, at $\omega_{0}=6$ the wavelet scale corresponds almost directly to the equivalent Fourier period (to within approximately 3%), simplifying the physical interpretation of the scalogram in terms of frequency [8].

**Implementation Parameters:**

- Logarithmic scale sampling: 32 voices per octave for dense frequency resolution.
- Time–frequency resolution adapted through fixed wavelet parameters.
- Power computation: energy representation via squared magnitude $P\left(t,f\right)={\left|W\left(a,b\right)\right|}^{2}$.

To enhance feature stability and reduce the dynamic range, a logarithmic compression is applied to the power scalogram:

(9) $$P_{\log}\left(t,f\right)=\log\left(1+P(t,f)\right)$$

Subsequently, the matrix is normalized using the L1 norm to yield a discrete probability distribution suitable for entropy computation:

(10) $$\tilde{P}\left(t_{i},f_{j}\right)=\frac{P_{\log}\left(t_{i},f_{j}\right)}{\sum_{i,j}P_{\log}\left(t_{i},f_{j}\right)}$$

Here the indices *i* and *j* denote, respectively, the discrete time-bin and frequency-bin positions of the time–frequency matrix, so that $\tilde{P}\left(t_{i},f_{j}\right)$ is the normalized power at the *i*-th time bin and *j*-th frequency bin.

This ensures the power representation behaves as a proper probability distribution over time–frequency bins.

#### 3.3.2. Synchrosqueezed Wavelet Transform

To improve spectral resolution, synchrosqueezing is applied to the wavelet coefficients. This post-processing technique reallocates wavelet energy to curves of instantaneous frequency, thus concentrating energy that would otherwise be spread across scale.

The instantaneous frequency at each scale–time coordinate is calculated from the temporal derivative of the phase of the wavelet coefficients:

(11) $$\omega_{\mathrm{inst}}\left(a,b\right)=-\frac{\partial }{\partial b}\arg\left\{W\left(a,b\right)\right\}$$

In practice, this derivative is computed numerically using finite differences.

The energy is reassigned by summing wavelet coefficients into the right instantaneous frequency bins:

(12) $$T\left(f,b\right)=\int W\left(a,b\right)\;\delta \left(\omega_{\mathrm{inst}}\left(a,b\right)-2\pi f\right)\frac{da}{a^{3/2}}$$

where δ is the Dirac delta function. In the discrete implementation (as used here), this is approximated through bin-based accumulation, mapping energy to discrete frequency bins derived from $\omega_{\mathrm{inst}}\left(a,b\right)$.

The synchrosqueezed time–frequency matrix preserves invertibility and energy conservation while offering improved localization of time-varying spectral features.

To ensure fair comparison between CWT and SS-CWT, the same wavelet basis (Morlet), octave resolution (32 voices per octave), and sampling rate (2000 Hz) were used. The synchrosqueezed power representation undergoes identical log-compression and L1 normalization as the CWT to prepare it for downstream entropy-based feature extraction.

### 3.4. Information-Theoretic Feature Extraction

#### 3.4.1. Time-Frequency Domain Entropy Measures

For each normalized time–frequency distribution $\tilde{P}\left(t_{i},f_{j}\right)$ derived from either standard or synchrosqueezed wavelet transformation, multiple entropy formulations quantify distributional characteristics:

**Global Shannon Entropy:**

(13) $$H_{\mathrm{Shannon}}=-\sum_{i,j}\tilde{P}\left(t_{i},f_{j}\right)\log_{2}\tilde{P}\left(t_{i},f_{j}\right)$$

This classical information measure quantifies average surprise or uncertainty across the complete time–frequency plane. Lower values indicate concentrated energy distributions (high predictability), while elevated values reflect dispersed, irregular spectral content.

**Frequency-Marginal (Spectral) Entropy:**

Integrating over time yields frequency-marginal probability:

(14) $$p_{\mathrm{freq}}\left(f_{j}\right)=\sum_{i}\tilde{P}\left(t_{i},f_{j}\right)$$

from which spectral entropy computes:

(15) $$H_{\mathrm{spectral}}=-\sum_{j}p_{\mathrm{freq}}\left(f_{j}\right)\log_{2}p_{\mathrm{freq}}\left(f_{j}\right)$$

This measure characterizes frequency domain diversity independent of temporal evolution, capturing bandwidth and spectral concentration properties.

**Time-Marginal (Temporal) Entropy:**

Conversely, frequency integration produces a temporal marginal:

(16) $$p_{\mathrm{time}}\left(t_{i}\right)=\sum_{j}\tilde{P}\left(t_{i},f_{j}\right)$$

yielding temporal entropy:

(17) $$H_{\mathrm{temporal}}=-\sum_{i}p_{\mathrm{time}}\left(t_{i}\right)\log_{2}p_{\mathrm{time}}\left(t_{i}\right)$$

This formulation quantifies temporal amplitude modulation complexity and rhythm irregularity.

**Rényi Entropy Family:**

The Rényi entropy generalizes Shannon’s formulation through order parameter α:

(18) $$H_{\mathrm{Rényi}}^{\left(\alpha \right)}=\frac{1}{1-\alpha }\log_{2}\sum_{i,j}{\left[\tilde{P}\left(t_{i},f_{j}\right)\right]}^{\alpha }$$

Different α values emphasize distinct distributional properties: α=0.5 (less than unity) amplifies contributions from rare events and low-probability regions, while α=2.0 (greater than unity) emphasizes dominant high-probability components while suppressing tail contributions. Both orders were computed to capture complementary aspects of the time–frequency distribution.

**Tsallis Non-Additive Entropy:**

The Tsallis formulation, parameterized by entropic index *q*, provides an alternative generalization:

(19) $$H_{\mathrm{Tsallis}}^{\left(q\right)}=\frac{1}{q-1}\left(1-\sum_{i,j}{\left[\tilde{P}\left(t_{i},f_{j}\right)\right]}^{q}\right)$$

Setting q=1.5 produces an entropy measure reflecting the principles of non-extensive statistical mechanics, potentially capturing long-range correlations in complex cardiac dynamics.

**Frequency-Band-Specific Entropy Analysis:**

The frequency axis was divided into four physiologically motivated sub-bands to detect localized spectral abnormalities characteristic of specific pathophysiological mechanisms:

- **Sub-band I (20–80 Hz):** Fundamental S1/S2 frequencies and low-frequency components.
- **Sub-band II (80–200 Hz):** First and second harmonic structure of normal heart sounds.
- **Sub-band III (200–400 Hz):** Primary murmur spectral content and mid-frequency turbulence.
- **Sub-band IV (400–800 Hz):** High-frequency components including split sounds and transient artifacts.

This division corresponds to the spectral physiology of heart sounds. Basic components of S1 and S2 are concentrated in the low frequencies while turbulent-flow murmurs and valvular transients reach progressively higher frequencies. Comparable physiologically motivated multi-band decompositions of phonocardiograms, with band edges closely matching those adopted here, have been used in prior PhysioNet/CinC-2016 studies [13,21], which supports the rationale for this scheme.

For each sub-band *k*, normalized probability distributions were constructed. This normalization ensures each sub-band matrix represents a valid probability distribution, satisfying the requirements for entropy-based feature extraction.

(20) $${\tilde{P}}_{k}\left(t_{i},f_{j}\right)=\frac{P(t_{i},f_{j})}{\sum_{f_{j}\in {\mathrm{band}}_{k}}\sum_{i}P\left(t_{i},f_{j}\right)},\;f_{j}\in {\mathrm{band}}_{k}$$

Shannon entropy and Rényi entropy (order α=2) were computed within each band, yielding eight additional band-specific features (4 bands × 2 entropy types). The complete per-window feature inventory is summarized in Table 2.

#### 3.4.2. Time-Domain Complexity Measures

Complementing time–frequency features, direct time-series analysis provides alternative complexity characterization:

**Permutation Entropy:**

This ordinal pattern-based measure quantifies temporal structure through symbolic dynamics. For embedding dimension *m* and time lag τ, the signal partitions into overlapping *m*-dimensional vectors. Each vector’s component ordering defines a permutation symbol π. The relative frequency of each permutation P(π) yields:

(21) $$H_{\mathrm{PE}}=-\sum_{\pi }P\left(\pi \right)\log_{2}P\left(\pi \right)$$

Implementation employed m=3 and τ=2, providing 3!=6 possible ordinal patterns.

**Sample Entropy:**

Sample entropy assesses signal self-similarity through template matching. For pattern length *m* and tolerance threshold *r*, the algorithm counts matches among subsequences of length *m* (denoted *B*) and extended length m+1 (denoted *A*). The measure computes as:

(22) $$\mathrm{SampEn}=-\ln\left(\frac{A}{B}\right)$$

Parameters m=2 and $r=0.15\times \sigma_{x}$ (where $\sigma_{x}$ is signal standard deviation) balance sensitivity and robustness.

**Spectral Entropy from Power Spectral Density:**

The power spectral density estimates across the analysis bandwidth (20–800 Hz) were generated using Welch’s periodogram method with Hamming window tapering. After normalization, frequency domain probability distribution was obtained for calculation of Shannon entropy.

**Envelope-Based Entropy:**

The amplitude envelope, extracted via Hilbert transformation, isolates the modulation structure of the signal, enabling complexity analysis independent of the carrier waveform. Both permutation entropy and sample entropy computed on the envelope characterize amplitude modulation complexity.

#### 3.4.3. Window-Level Training with Recording-Level Evaluation

Instead of aggregating the window-level features into recording-level statistical summaries, we employ a multiple instance learning paradigm where the classifier operates directly on the individual window feature vectors. We use this approach to preserve temporal variability within recordings and to allow assessment at the recording level. Each 2 s window yields a feature vector $f_{w}\in R^{d}$ comprising the entropy measures described above. During training, windows are treated as independent samples, whereas, at evaluation, predictions from all windows within a recording are aggregated via probability averaging:

(23) $${\hat{p}}_{r}=\frac{1}{N_{r}}\sum_{w=1}^{N_{r}}p\left(y=1|f_{w}\right)$$

where $p(y=1|f_{w})$ is the predicted probability of abnormality for window *w*. This aggregation approach generates a prediction at the recording level, while leveraging the temporal variability within a recording captured by the window-level model. There are many advantages to this method over feature-level aggregation:

- Preservation of within-recording temporal patterns that may be lost in statistical summaries.
- Elimination of the feature dimension explosion inherent in computing multiple statistics per feature.
- Natural handling of variable-length recordings without padding or truncation artifacts.

### 3.5. Classification Architecture

#### 3.5.1. Neural Network Model

A compact multi-layer perceptron (MLP) architecture was designed to provide nonlinear classification capability based on extracted entropy features.

**Layer Structure:**

- **Input layer:** Dimensionality matching the recording-level entropy feature vector (ranging from 40 to 150 features depending on time–frequency representation and entropy configuration).
- **First hidden layer:** 128 neurons with ReLU activation, followed by dropout (probability 0.3).
- **Second hidden layer:** 64 neurons with ReLU activation, followed by dropout (probability 0.3).
- **Output layer:** Single neuron with sigmoid activation.

**Objective Function:**

Binary cross-entropy loss with class-imbalance weighting:

(24) $$L\left(\theta \right)=-\frac{1}{N}\sum_{n=1}^{N}w_{y_{n}}\left[y_{n}\log{\hat{y}}_{n}\left(\theta \right)+\left(1-y_{n}\right)\log\left(1-{\hat{y}}_{n}\left(\theta \right)\right)\right]$$

where $y_{n}\in \left\{0,1\right\}$ denotes the ground truth label, ${\hat{y}}_{n}\left(\theta \right)$ represents the predicted probability from the model with parameter θ, and $w_{y_{n}}$ applies inverse class frequency weighting.

**Optimization Protocol:**

The model was trained using the Adam optimizer with an initial learning rate $\eta =10^{-3}$ and mini-batch size of 32. To prevent overfitting early stopping was used with a patience of 20 epochs, and also, learning rate was halved upon plateau in validation performance.

**Regularization:**

While model training, to control the model complexity, dropout (p=0.3) was applied after each hidden layer, and L2 weight regularization was also used with coefficient $\lambda =10^{-4}$.

#### 3.5.2. Baseline Comparison Models

Baseline classifiers include logistic regression with L2 regularization and XGBoost, both trained on the same recording-level feature vectors. Hyperparameters for XGBoost were tuned via grid search on the training fold to ensure fair comparison.

### 3.6. Experimental Validation Protocol

#### 3.6.1. Cross-Validation Strategy

Stratified 5-fold cross-validation with recording-level partitioning was employed to prevent data leakage between folds. To guide early stopping, within each fold, the training split was further subdivided into 90% training and 10% validation sets.

#### 3.6.2. Performance Metrics

Due to the inherent class imbalance in clinical PCG datasets, evaluation was performed using both overall and balanced accuracy.

Accuracy is the overall proportion of correct predictions but can be misleading in imbalanced dataset setups. Balanced accuracy (BAcc) gives an agnostic metric with respect to classes by averaging recall over Normal and Abnormal classes. This way, the prevalence of the dominant class does not affect the detection of minority classes, and we obtain a more robust and clinically meaningful evaluation. Primary Metric will be Balanced Accuracy:

(25) $$\mathrm{BAcc}=\frac{1}{2}\left(\frac{TP}{TP+FN}+\frac{TN}{TN+FP}\right)$$

Secondary Metrics for recording-level predictions are evaluated using:

- Area Under ROC Curve (AUC): Primary metric for ranking performance.
- Sensitivity and Specificity: Clinical interpretability at multiple operating points:
  – Threshold = 0.5 (default);
  – Youden’s index: $\arg\max_{\theta }\left(Sensitivity\left(\theta \right)+Specificity\left(\theta \right)-1\right)$;
  – A 90% specificity threshold (clinically conservative).
- F1-score, Precision, Accuracy: Complementary performance indicators.

Metrics are reported as mean ± standard deviation across 5-fold CV, with final performance assessed on the held-out test set.

### 3.7. Ablation Study Design

A systematic ablation study was conducted to quantify the contribution of key components in the classification pipeline:

- Type of time–frequency representation (CWT vs. SS-CWT).
- Entropy feature composition (global, band-specific, temporal).
- Frequency bandwidth (600 Hz vs. 800 Hz upper limit).

### 3.8. Implementation and Reproducibility

All experiments were conducted in Python 3.9 using the following package versions: SciPy 1.9.3, PyWavelets 1.4.1, ssqueezepy 0.6.3, scikit-learn 1.2.0, TensorFlow 2.11.0, NumPy 1.23.5, and pandas 1.5.2. Fixed random seeds (value: 42) ensured reproducible results across all training runs and folds.

All experiments, including the reported timing measurements, were executed on an Apple M3 Max system, i.e., 14-core CPU comprising 10 performance and 4 efficiency cores, 36 GB unified memory, running macOS 26. To ensure deterministic and reproducible behavior, neural-network training and inference were configured to run on the CPU in single-threaded mode, with GPU and Neural Engine acceleration disabled, feature extraction was likewise performed on the CPU. No GPU acceleration was used for any reported result, so the timing figures reflect a conservative, commodity CPU-only execution.

The reported end-to-end processing time of approximately 1.2 s per recording, including feature extraction and classification combined, corresponds to the optimal configuration (CWT, Global + Band + TD, 20–800 Hz) on the hardware described above. This cost is dominated by the time–frequency transform and the subsequent entropy computation. The multilayer-perceptron inference is negligible by comparison, as it applies a small dense network to a 20-dimensional feature vector. Consequently, the processing time is largely insensitive to the size of the entropy feature set, because the global, band-wise, and time-domain features are all derived from a single time–frequency matrix that is computed once per window and reused. The principal factor governing computational cost is the choice of transform: the synchrosqueezed CWT incurs additional overhead relative to the standard CWT because of the instantaneous-frequency reassignment step. The recommended CWT-based configuration is therefore also the more computationally efficient one, so the accuracy and efficiency objectives are aligned rather than in conflict.

## 4. Results

### 4.1. Experimental Design

All experiments were performed using five-fold stratified cross-validation at the recording level. Performance was evaluated by training at the window level and testing at the recording level through averaging of probabilities. The final performance validation was provided by a held-out test set composed of 15% of the recordings. This research work conducted extensive ablation studies to systematically evaluate the discriminative ability of different time–frequency representations and entropy feature configurations across:

- Time-Frequency Representation: Continuous Wavelet Transform (CWT) vs. Synchrosqueezed CWT (SSQ).
- Entropy Feature Sets: Global (6 features), Global + Bandwise (12–14 features), Global + Bandwise + Time-Domain (18–20 features).
- Frequency Bandwidth: 20–600 Hz (excluding band IV) vs. 20–800 Hz (full spectrum).

This yielded 12 experimental configurations, each evaluated with identical neural network architecture (128-64-1 MLP with batch normalization and dropout) and training protocols.

### 4.2. Primary Results: Optimal Configuration

The highest classification performance was achieved using CWT-derived features with the complete entropy feature set (Global + Bandwise + Time-Domain) across the full 20–800 Hz bandwidth. Table 3 summarizes the performance of this configuration. Here, the optimal configuration refers to the transform and entropy feature set that attained the highest mean five-fold cross-validation AUROC during model selection, the values reported in this table are the performance of that configuration on the independent held-out test set.

The model demonstrated good stability across cross-validation folds with AUROC SD of 0.007 and achieved test AUROC of 0.972 with balanced accuracy of 0.915 at Youden’s optimal threshold. At the clinically conservative operating point of 90% specificity, the model achieved 95% sensitivity, which is appropriate for screening applications where false negatives carry higher clinical cost.

### 4.3. Ablation Study I: Time-Frequency Representation

To evaluate the impact of spectral reassignment on classification performance, we compared CWT against its synchrosqueezed variant (SS-CWT) across all feature configurations. Table 4 presents this comparison. In this table and the subsequent ablation tables, “Global” denotes entropy measures computed over the entire time–frequency plane only (6 features) and does not include any frequency-band-specific features, “Global + Band” additionally includes the band-wise entropies, and “Global + Band + TD” further adds the time-domain features.

Key Finding: CWT outperformed SS-CWT in all configurations compared to theoretical expectations. The performance difference was most pronounced for sparse feature sets (Global only: Δ=+0.085 AUROC), and dipped with increasing feature richness (Global + Band + TD: Δ=+0.006 to +0.013). This odd outcome suggests several possible explanations:

1. Spectral Smearing as Feature Richness: The intrinsic frequency spreading in CWT can capture wider harmonic relations between overlapping cardiac components (S1/S2/murmurs) and thus provide more robust features than the localized energy distributions of SS-CWT.
2. Reassignment Sensitivity: SS-CWT’s aggressive energy reassignment may be vulnerable to transient artifacts common in heterogeneous PCG recordings, potentially discarding diagnostically relevant spectral variability.
3. Entropy Interaction: The various entropy measures, like Shannon, Rényi, Tsallis, may respond differently to the different statistical characteristics of CWT versus SS-CWT distributions. Shannon entropy may favor the smoother CWT representation.

While SS-CWT is more localized in time–frequency, the classification task benefits more from a full spectral characterization of CWT. This finding has practical implications where CWT is less computationally demanding than SS-CWT and yet achieves better performance.

### 4.4. Ablation Study II: Entropy Feature Contributions

Better performance improvements were observed when entropy feature categories were added progressively, indicating the complementary nature of different information-theoretic measures. Table 5 quantifies this progression for the optimal TFR (CWT, 800 Hz).

The most substantial improvement (+0.038 AUROC, +4.1%) resulted from incorporating frequency-band-specific entropy measures, indicating that localized spectral irregularities carry significant diagnostic information beyond global time–frequency statistics. Specifically, the four physiologically motivated sub-bands (20–80 Hz: S1/S2 fundamentals; 80–200 Hz: harmonics; 200–400 Hz: murmurs; 400–800 Hz: transients) capture distinct pathological signatures that aggregate entropies obscure.

Time-domain complexity measures (permutation entropy, sample entropy, spectral entropy on raw signal and envelope) provided additional gains (+0.007 AUROC), confirming that purely temporal dynamics complement but do not replace time–frequency analysis. This modest contribution suggests that wavelet-based features already capture most temporally-encoded information. Cross-Validation Stability: Introducing bandwise features greatly enhanced the model consistency (AUROC SD: 0.030 → 0.006), suggesting that local spectral characterization results in more robust classification across different recording conditions in the PhysioNet 2016 dataset.

### 4.5. Ablation Study III: Frequency Bandwidth

Extending the analysis bandwidth from 600 Hz to 800 Hz yielded consistent, modest performance improvements across all configurations (Table 6).

The 400–800 Hz band included diagnostic information beyond a traditional stethoscope, particularly when combined with bandwise entropy analysis. This high-frequency content likely reflects the following.

- Transient features such as split sounds like S2 splitting in bundle branch blocks.
- Fine temporal structure of valve closure events.
- High-frequency components of certain murmurs, e.g., aortic regurgitation diastolic murmur.

It is clear that global entropy features showed no improvement with extended bandwidth (Gain = 0.000), confirming that the benefit is derived specifically based on band-localized analysis rather than mere spectral extension.

### 4.6. Baseline Comparisons

To contextualize the neural network’s performance, we compared it against two conventional machine learning baselines trained on identical window-level features: (1) L2-regularized logistic regression with class-balanced weights, and (2) XGBoost with 400 trees and maximum depth of 4, with a learning rate of 0.05. Table 7 presents comparative results for the optimal configuration.

Based on the comparative results the analysis is:

- The MLP and XGBoost achieved the same AUROC of 0.972, and both significantly outperformed logistic regression (0.907, Δ=0.065).
- The MLP demonstrated better balanced accuracy (0.915 vs. 0.882) because of high sensitivity (0.960) with reasonable specificity (0.871).
- XGBoost traded off sensitivity (0.800) for very high specificity (0.964), reflecting different decision boundary geometries.
- For screening clinical cases where false negatives (missed abnormalities) carry higher cost, the MLP’s operating point (96% sensitivity, 87% specificity) is preferable to XGBoost’s (80% sensitivity, 96% specificity). An increase in sensitivity of 16 percentage points is equivalent to 16 more abnormal patients detected in 100 patients.
- The higher F1-score obtained by XGBoost (0.825 versus 0.780) does not indicate superior screening ability. F1 is an operating-point-dependent measure that, under the class imbalance of the test set, rewards the higher precision of XGBoost (approximately 0.85 versus 0.65 for the MLP). This precision is itself a consequence of its conservative, high-specificity decision threshold, which suppresses false positives at the cost of missing roughly one in five abnormal recordings. Because both classifiers attain identical threshold-independent discrimination (AUROC = 0.972), the F1 difference reflects the chosen operating point rather than any genuine difference in discriminative power.

Accordingly, the MLP is preferred not because it dominates on every individual metric, but because, at equivalent overall discrimination, it provides the operating point best aligned with screening priorities, namely the highest sensitivity (0.960) and balanced accuracy (0.915) among the compared methods. For this reason, threshold-independent AUROC and sensitivity, rather than F1, are emphasized as the primary basis for model selection in this screening context.

Table 8 extends this comparison across all 12 experimental configurations.

Key observations:

- The MLP achieved best of AUROC in 10 of 12 configurations.
- XGBoost matched or outperformed MLP only with rich feature sets, i.e., Global + Band or Global + Band + TD).
- The MLP’s strength was most apparent for sparse features (Global-only: +0.116 vs. LogReg), suggesting that the neural architecture compensates for feature sparsity through learned representations.
- Logistic regression was consistently worse (average 0.852), suggesting that linear decision boundaries are not sufficient.

### 4.7. Visual Performance Analysis

**Figure 2:**
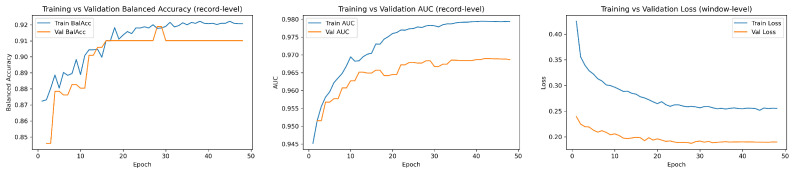
Training and validation curves for optimal configuration (CWT, 800 Hz, Global + Band + TD). Balanced accuracy, AUROC, and binary cross-entropy loss demonstrate stable convergence.

**Figure 3:**
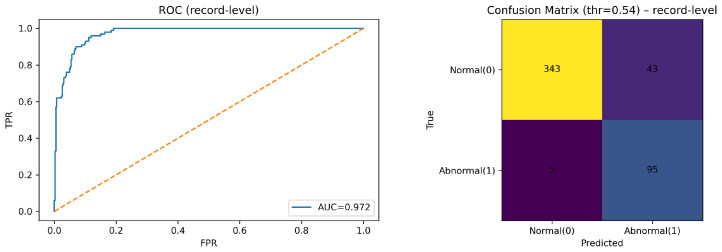
Test set evaluation: (**Left**) Blue line presents the ROC curve with AUROC = 0.972, and the dashed orange line presents a classifier baseline (**Right**) Confusion matrix at Youden’s threshold showing 96% sensitivity and 87% specificity.

Figure 2 presents training and validation curves for the optimal configuration, confirming convergence without overfitting. Figure 3 displays the test ROC curve (AUROC = 0.972) and confusion matrix at Youden’s optimal threshold.

**Figure 4:**
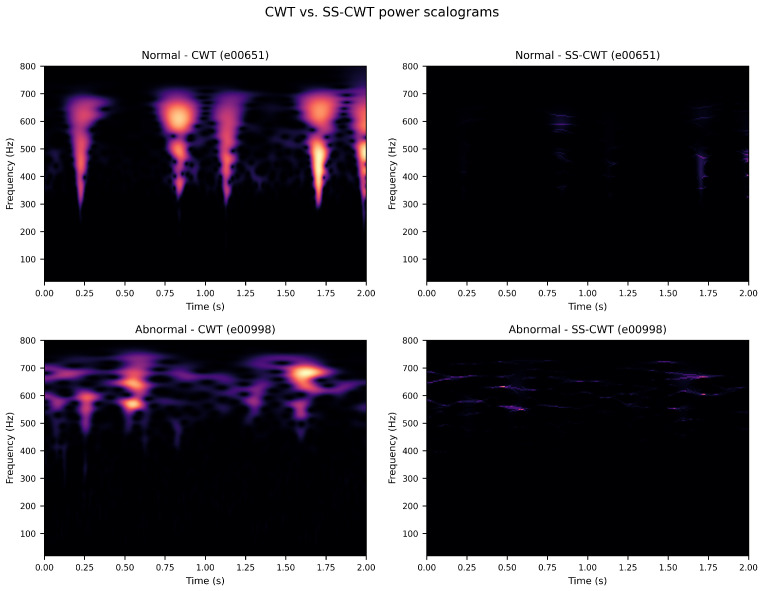
CWT versus SS-CWT power scalograms for a representative normal (**top**) and abnormal (**bottom**) recording. Synchrosqueezing concentrates the energy into a few sparse ridges and discards much of the distributed energy retained by the CWT, illustrating why the entropy descriptors are more discriminative under the standard CWT.

**Figure 5:**
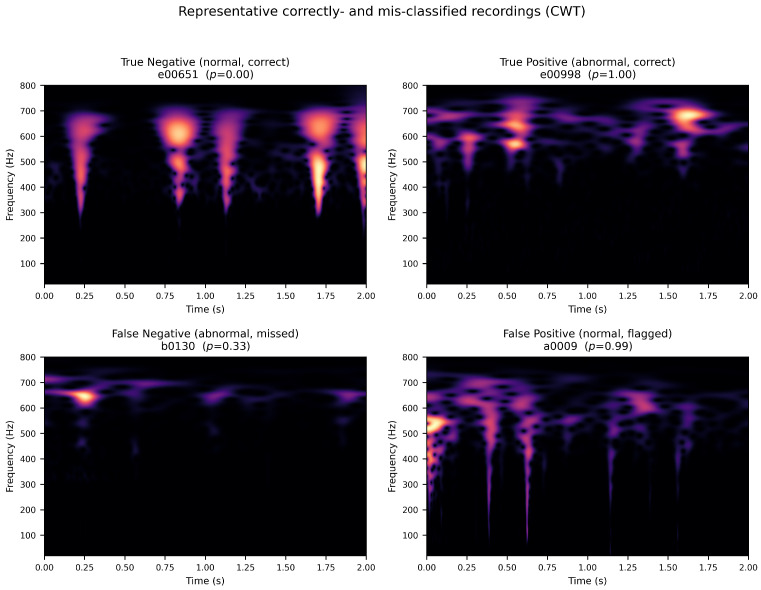
Representative CWT scalograms for the four outcome categories on the test set: a correctly classified normal (true negative) and abnormal (true positive) recording, a missed abnormal (false negative), and an over-flagged normal (false positive). The false negative exhibits subtle, narrow-band energy resembling a normal pattern, whereas the false positive shows broadband, noise-like energy extending to low frequencies.

**Figure 6:**
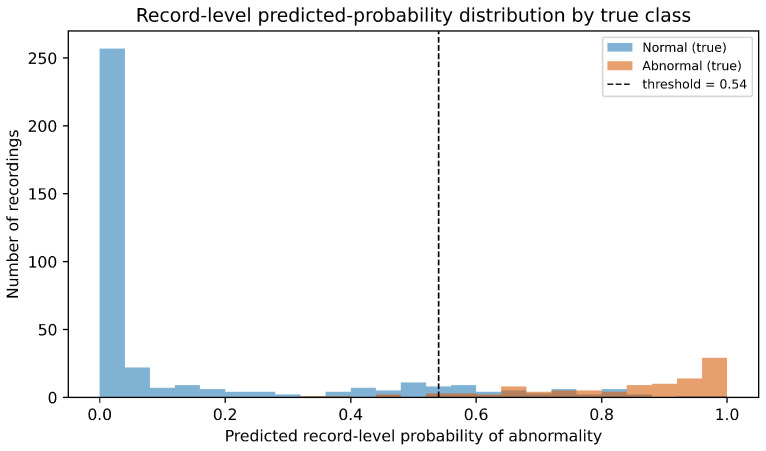
Distribution of record-level predicted probabilities on the test set, separated by true class. Normal recordings concentrate near zero and abnormal recordings near one, with the residual overlap around the decision threshold accounting for the misclassified cases.

To provide a more intuitive account of model behavior and to examine the sources of error, three further analyses are presented: Figure 4 contrasts the CWT and SS-CWT power scalograms for a representative normal and abnormal recording, Figure 5 shows representative correctly and misclassified recordings and Figure 6 shows the distribution of record-level predicted probabilities by true class.

The error analysis reveals a clear and clinically favorable structure. Among the 486 test recordings, 48 were misclassified at the model’s operating threshold, which comprises of 43 false positives and only 5 false negatives, which is consistent with the high-sensitivity operating point of the screening model. All five missed abnormal recordings were borderline rather than confident errors, with predicted probabilities between 0.33 and 0.53, which was just below the 0.54 threshold. Four of them were from a single acquisition source. As shown in Figure 5, these missed cases tend to present subtle, narrow-band energy that visually resembles normal heart sounds. Most false positives were similarly near-threshold, whereas 22 of 43 had predicted probability below 0.7. The smaller number of confident false positives correspond to recordings with broadband, which is noise-like energy that mimics the spectral spread of pathology. The errors were not distributed uniformly across the dataset acquisition sources but concentrated in a subset of them, reinforcing that recording conditions and acquisition site are an important source of residual error and a further motivation for the multi-site validation discussed in the limitations.

## 5. Discussion

### 5.1. Unexpected CWT Superiority over SS-CWT

The most important result of this study is the better performance of the standard CWT in comparison to the theoretically better SS-CWT. While synchrosqueezing concentrates the energy along instantaneous frequency trajectories, this sharpening may inversely reduce the distinctive capacity for several reasons:

1. Harmonic Information Loss: Pathological heart sounds often shows diffuse harmonic structures, e.g., systolic murmurs with wide spectral content. CWT’s spectral smearing may be more effective in preserving inter-harmonic relationships broken apart by SS-CWT’s reassignment.
2. Robustness to Artifacts: The PhysioNet 2016 database contains recordings from various sources with different noise characteristics. CWT’s smoother representation may be more sensitive to transient artifacts that could fool SS-CWT’s reassignment algorithm.
3. Entropy-Specific Advantages: Shannon and Rényi entropies are measures of spread in distribution. The larger energy distributions of CWT may yield more discriminating entropy values than the localized peaks of SS-CWT, especially for the computation of bandwise entropies where localized irregularities dominate.

This finding suggests that for entropy-based classification, representation smoothness may be more valuable than time–frequency precision, contrasting with applications, e.g., signal reconstruction, mode separation, where SS-CWT excels. This interpretation is visually corroborated by Figure 4: under SS-CWT the scalograms collapse to a few sparse ridges, whereas the CWT retains the broader, distributed energy on which the entropy descriptors operate.

### 5.2. Value of Multi-Scale Entropy Analysis

The improvement from bandwise entropy with +4.1% AUROC confirms that cardiac pathologies exist at specific frequency ranges corresponding to distinct physiological mechanisms. The four-band decomposition effectively isolates:

- **Band I (20–80 Hz):** Valve timing abnormalities (S1/S2 splitting, extra sounds).
- **Band II (80–200 Hz):** Harmonic distortion in murmurs.
- **Band III (200–400 Hz):** Turbulent flow signatures.
- **Band IV (400–800 Hz):** High-frequency transients and valve click details.

This localized analysis aligns with clinical auscultation practice, where physicians mentally filter different frequency ranges during examination.

In addition to the frequency band choice, the individual entropy measures provide complementary information because each one is mathematically sensitive to a different aspect of the time–frequency distribution. Shannon entropy measures the overall dispersion of spectral energy, and increases as the distribution becomes more uniform. Different order Rényi entropies probe different parts of this distribution: low order (α=0.5) amplifies the contribution of low probability bins and is therefore sensitive to the diffuse, broadband energy of turbulent murmurs, while high order (α=2) is dominated by the most energetic bins and reflects the concentrated energy of normal S1/S2 complexes. The Tsallis entropy is non-extensive and therefore also responsive to correlations in the distribution rather than treating bins as independent. Spectral entropy measures spectral flatness and distinguishes the tonal, peaked spectra of normal sounds from the noise-like, flatter spectra produced by murmurs, whereas permutation and sample entropies work in the time domain and capture, respectively, the complexity of ordinal temporal patterns and the regularity of the waveform.

Physiologically, normal heart sounds are quasi-periodic, with energy concentrated in well defined low-frequency bands, yielding low and stable entropy values. Pathological sounds instead introduce diffuse, irregular, broadband energy that raises entropy in a measure- and band-dependent manner. Because each metric emphasizes a distinct facet of this change, e.g., low-probability tails versus dominant peaks, spectral flatness versus temporal regularity, these measures are complementary rather than redundant. This explains why their combination performs better than any single descriptor and the results are consistent with the incremental gains observed in the entropy ablation study.

### 5.3. Clinical Significance and Practical Application

The clinical utility of the proposed method is to provide a consistent and unbiased screening aid for cardiac auscultation. Auscultation with a stethoscope still remains the primary and lowest-cost method of cardiac assessment, but its reliability is strongly dependent on the experience of the examiner and significant inter-observer disagreement has been documented [2]. Echocardiography, which is a standard diagnostic reference, is itself operator-dependent, where it requires a specialized equipment and expertise not always available at the point of care or in resource-limited settings. In addition, image quality may be degraded in patients with obesity or pulmonary emphysema, whereas, an automated phonocardiogram classifier is a trade off between these two extremes where it provides a reproducible, inexpensive assessment step that is independent of the skill of the interpreter.

In practical terms, the method is intended to function as a decision-support and triage tool, not as a replacement for clinical judgment or for definitive imaging. A short heart-sound recording, acquired with a digital stethoscope at a standard auscultation site, is processed in approximately 1.2 s on commodity CPU hardware, with the trained network itself producing a prediction in a few milliseconds, and returns a normal vs. abnormal probability. Recordings flagged as abnormal can be prioritized for clinician review and onward referral for echocardiography or specialist evaluation, while recordings classified as normal support routine follow-up. Because the classification pipeline can run on a standard laptop or mobile device without specialized hardware, it is well suited to primary-care, community-screening, and telemedicine contexts in which access to cardiology expertise is limited.

Two properties are particularly relevant to this screening role. First, the chosen operating point favors high sensitivity (96%, and 95% at 90% specificity), so that few abnormal recordings are missed, where in a screening context the cost of a false negative, i.e., a missed abnormality, generally outweighs that of a false positive, an unnecessary referral, which makes this trade-off clinically appropriate. Second, the features are physiologically interpretable: because entropy is computed within defined frequency bands, an abnormal result can be linked to the band in which irregular spectral energy arises (for example, energy in the murmur-related band), which is more transparent to a clinician than the output of an opaque deep network.

It must be emphasized that these are statements of intended clinical use and potential value rather than of demonstrated clinical performance. The method distinguishes only normal from abnormal heart sounds, does not identify specific pathologies, and has not yet undergone prospective clinical validation. Establishing its diagnostic accuracy in real-world practice, and comparing it directly against clinician auscultation, are the objectives of the validation program described in the Limitations and Future Work below.

### 5.4. Limitations and Future Work

Several limitations should be acknowledged. First, and most importantly, all training and evaluation were conducted within a single benchmark corpus. Although the PhysioNet/CinC-2016 database aggregates recordings from several acquisition sources, this within-corpus diversity does not establish that the method generalizes to entirely independent cohorts: performance under distribution shift to new acquisition devices, clinical sites, patient populations (e.g., pediatric subjects), or noise environments has not been verified and should not be assumed from the pooled-source results reported here. Robust deployment at a previously unseen site would therefore be expected to require site-specific recalibration or domain adaptation, and external multi-site validation is a prerequisite before clinical use. Second, the present framework addresses only the binary normal vs. abnormal task and does not differentiate specific pathologies such as stenosis or regurgitation, which would require datasets with richer pathology-level annotations. Third, although the entropy features are individually interpretable, the multilayer-perceptron decision process itself remains comparatively opaque.

Future work will pursue three directions. (i) External and multi-site validation on independent corpora (for example, the CirCor DigiScope 2022 and PASCAL databases) and on prospectively collected clinical recordings, together with domain-adaptation and per-site calibration techniques to address cross-site distribution shift. (ii) Extension from binary screening to multi-class classification of specific valvular pathologies and to murmur grading, contingent on suitably annotated data. (iii) Prospective comparison of the method against expert auscultation in a clinical setting, in order to quantify its added diagnostic value and to establish its diagnostic accuracy under real-world conditions.

## 6. Conclusions

This research work established a computationally efficient methodology for automated phonocardiogram classification integrating wavelet-based time–frequency analysis with information-theoretic feature extraction. Evaluation on the PhysioNet/Computing in Cardiology Challenge 2016 dataset with 3240 recordings demonstrated that the optimal configuration continuous wavelet transform at 800 Hz bandwidth with multi-scale entropy features achieved a test set AUROC of 0.972, balanced accuracy of 0.915, and 96% sensitivity at 90% specificity, matching state-of-the-art deep learning approaches while using substantially fewer features (20 vs. thousands of parameters). Systematic ablation studies yielded three principal insights:

- Standard CWT consistently outperformed synchrosqueezed CWT with average AUROC advantage of +0.038, indicating spectral smoothing captures diagnostically relevant harmonic relationships better than aggressive frequency localization.
- Band specific entropy analysis provided the largest performance contribution of +4.1% in AUROC, confirming that cardiac pathologies manifest unique signatures across physiologically motivated frequency bands.
- Extending bandwidth range to 800 Hz has captured high-frequency transient features that are beyond the range of traditional stethoscope.

The methodology meets clinical screening requirements with 1.2 s feature extraction, enabling real-time care deployment and interpretable entropy-based features and supporting clinician trust. However, limitations include dataset-specific validation requiring external generalization studies, binary classification frameworks are unable to distinguish specific pathology differentiation, and neural networks are opaque but the input features interpretable.

## Figures and Tables

**Table 1:** Models reported in the literature regarding PCG classification (AUROC primary; BalAcc: Balanced Accuracy; Se: Sensitivity; Sp: Specificity; NR: not reported).

Author	Dataset & Splits	Model/Features	Results
Proposed method	PhysioNet/CinC Challenge 2016; 5-fold record-level	MLP/entropy from CWT (20–800 Hz) 2 s with 1 s overlapping window	AUROC 0.972; BalAcc 0.915; Se@90%Sp ≈ 0.92.
Vieira et al. 2023 [10]	CinC 2016 (set A); 70/30 stratified record-wise split; 5-fold CV on train	5 s CWT scalograms → CNN (VGG-16 variants); transfer learning (ImageNet; ECG-2017; PCG b–f)	AUROC 0.913; Acc 0.828; Rec 0.931; Prec 0.844; F1 0.885; Sp: NR
Goda et al. 2016 [11]	CinC 2016 (official hidden test; LR–HSMM segmentation; 20–400 Hz; 1 kHz)	Time + frequency (systolic/diastolic spectral bands) + DWT envelopes (homomorphic); SVD → SVM	AUROC: NR; Se 83.77%; Sp 76.8%; MAcc 80.28%
Zabihi et al. 2016 [12]	CinC 2016 (Challenge); segmentation-free; quality → anomaly cascade	18/40 selected handcrafted features (LPCs; Shannon/Tsallis entropy; MFCC stats; DWT (db4) band entropies/variance; PSD centroid/band AUCs); ensemble of 20 FF-ANNs (committee)	AUROC: NR; Se 86.91%; Sp 84.90%; MAcc 85.90%
Potes et al. 2016 [13]	CinC 2016; in-house 80/20 (record-wise) + official blind test; LR–HSMM segmentation; 1 kHz; 25–400 Hz	124 time/freq features (intervals, amplitude stats, 9 band powers per state, MFCCs) → AdaBoost-abstain; 4-band cycle CNN (25–45/45–80/80–200/ 200–400 Hz) with OR-rule ensemble	AUROC: 0.91 (AdaBoost), 0.92 (CNN); Se 0.9424, Sp 0.7781, MAcc 0.8602
Langley et al. 2016 [14]	CinC 2016; 5 s unsegmented; CWT (Gaus4); scale 1.7; threshold 7.8	Wavelet entropy (single threshold classifier)	Se 98%, Sp 56%, MAcc 77%
Singh-Miller et al. 2016 [15]	CinC 2016 (Challenge); segmentation-free; STFT spectrograms (Gaussian win 15/75)	Band-wise spectral stats + logistic-fit params; feature selection; Random Forest	Se 0.76; Sp 0.87; MAcc 0.81; CV score ≈ 0.85
Taneja et al. 2023 [19]	PhysioNet/CinC-2016; single 80/20 hold-out (record grouping N/S)	SVM (RBF) vs. LSSVM, k-NN, DT, ANN & LBP/ALBP/RLBP on spectrogram + chromagram fusion	Acc 0.9212; Se 0.9753; Sp 0.6859; BalAcc 0.874
Marocchi et al. 2023 [21]	CinC-2016 (training-a); 60/20/20 split; LR–HSMM; 1 kHz; PCG 25–400 Hz; ECG 2–60 Hz; 10 cycles/patient; PCG split into 4 bands	Spectrogram/Mel/SS-CWT (Morlet) images → transfer learning (ResNet, VGG, InceptionV3); PCG + ECG ensemble; XAI (Grad-CAM, GBP, LIME)	Best (InceptionV3, spectrogram, PCG(4) + ECG): Acc 91.25%; Se 98.33%; Sp 70.00%
Huang et al. 2025 [22]	Echocardiography + clinical data (nine categories); hospital datasets; 70/30 split + 10-fold CV (imbalanced)	XGBoost, LightGBM, CATBoost, RF; RFE + voting ensemble and Top features after RFE: Age, AO, LV, LA	Acc: 0.843 (XGB), 0.884 (CATBoost);

**Table 2:** Entropy feature inventory per window.

Feature Category	Transform	Count
Global (Shannon, Spectral, Temporal, Rényi × 2, Tsallis)	CWT/SSQ	6
Bandwise (Shannon, Rényi) × 4 bands (20–800 Hz)	CWT/SSQ	8
Time-domain (Perm, SampEn, SpecEnt) × (raw, envelope)	-	6

**Table 3:** Optimal Configuration Performance (CWT, 800 Hz, Global + Band + TD, 20 features).

Metric	5-Fold CV	Test @ 0.50	Test @ Youden
Balanced Accuracy	0.881 ± 0.013	0.901	**0.915**
AUROC	0.958 ± 0.007	**0.972**	**0.972**
F1-Score	0.717 ± 0.019	0.754	0.780
Sensitivity	0.936 ± 0.037	0.950	0.960
Specificity	0.826 ± 0.028	0.852	0.871
Precision	0.583 ± 0.031	0.625	0.658
Accuracy	0.848 ± 0.016	0.872	0.889
Sens.@90%Spec	0.850 ± 0.029	0.890	0.920

**Table 4:** CWT vs. SS-CWT performance comparison (Test AUROC).

Entropy Set	Bandwidth	CWT	SS-CWT	Δ (CWT − SSQ)
Global	600 Hz	0.927	0.842	**+0.085**
Global	800 Hz	0.927	0.842	**+0.085**
Global + Band	600 Hz	0.955	0.932	+0.023
Global + Band	800 Hz	0.965	0.946	+0.019
Global + Band + TD	600 Hz	0.968	0.955	+0.013
Global + Band + TD	800 Hz	**0.972**	0.966	+0.006
**Average**		**0.952**	0.914	**+0.038**

**Table 5:** Feature set ablation study (CWT, 800 Hz).

Feature Set	Features	CV AUROC	Test AUROC	Gain
Global Only	6	0.884 ± 0.010	0.927	–
Global + Bandwise	14	0.950 ± 0.006	0.965	**+0.038**
Global + Band + TD	20	0.958 ± 0.007	**0.972**	+0.007

**Table 6:** Frequency range comparison (Test AUROC).

TFR	Entropy Set	600 Hz	800 Hz	Gain
CWT	Global	0.927	0.927	0.000
CWT	Global + Band	0.955	0.965	+0.010
CWT	Global + Band + TD	0.968	**0.972**	+0.004
SS-CWT	Global	0.842	0.842	0.000
SS-CWT	Global + Band	0.932	0.946	+0.014
SS-CWT	Global + Band + TD	0.955	0.966	+0.011

**Table 7:** Method comparison: optimal configuration (CWT, 800 Hz, Global + Band + TD).

Method	AUROC	Bal. Acc.	Sens.	Spec.	F1
Logistic Regression	0.907	0.863	0.930	0.795	0.684
XGBoost (400 trees)	**0.972** *	0.882	0.800	**0.964**	0.825
**MLP (Proposed)**	**0.972**	**0.915**	**0.960**	0.871	0.780

* XGBoost matches MLP AUROC but trades sensitivity for specificity.

**Table 8:** Comprehensive baseline comparison: test AUROC across all configurations.

TFR	Features	LogReg	XGBoost	MLP	Δ vs. Best Baseline
CWT, 600 Hz					
	Global (6)	0.811	0.913	**0.927**	+0.014
	Global + Band (12)	0.882	0.955	**0.955**	0.000
	Global + Band + TD (18)	0.892	0.970	0.968	−0.002
CWT, 800 Hz					
	Global (6)	0.811	0.913	**0.927**	+0.014
	Global + Band (14)	0.888	0.966	0.965	−0.001
	Global + Band + TD (20)	0.907	**0.972**	**0.972**	0.000
SS-CWT, 600 Hz					
	Global (6)	0.776	0.851	**0.842**	−0.009
	Global + Band (12)	0.832	0.912	**0.932**	+0.020
	Global + Band + TD (18)	0.872	0.936	**0.955**	+0.019
SS-CWT, 800 Hz					
	Global (6)	0.776	0.851	**0.842**	−0.009
	Global + Band (14)	0.873	0.936	**0.946**	+0.010
	Global + Band + TD (20)	0.890	0.952	**0.966**	+0.014
Average across all configs		0.852	0.931	**0.941**	**+0.010**

## Data Availability

The original data presented in the study are openly available in the PhysioNet repository: https://physionet.org/content/challenge-2016/1.0.0/ (accessed on 1 May 2026). The dataset was published as part of “The PhysioNet/Computing in Cardiology Challenge 2016” [24] and is referenced in the Section 3.1.
